# Supplementary material for: Nanoemulsion-directed growth of MOFs with versatile architectures for the heterogeneous regeneration of coenzymes
Source: Nat Commun. 2022 Apr 6;13:1879. doi: 10.1038/s41467-022-29535-7 (PMC8986779; doi:10.1038/s41467-022-29535-7)
Supplement: Supplementary file 1 — Supplementary Information [file 41467_2022_29535_MOESM1_ESM.pdf]

Supplementary Information

for

Nanoemulsion-directed growth of MOFs with versatile architectures for the heterogeneous regeneration of coenzymes

Ke Li, Yucheng Zhao, Jian Yang, and Jinlou Gu*

Key Laboratory for Ultrafine Materials of Ministry of Education, School of Materials Science and Engineering, East China University of Science and Technology, Shanghai 200237, China

*E-mail: jinlougu@ecust.edu.cn. Fax: +86-21-64250740. Tel: +86-21-64252599 **(J.Gu)**

Supplementary Methods

1. Chemicals and Materials

Zirconium (IV) oxynitrate dihydrate ($ZrO(NO_3)_2 \cdot 2H_2O$), L-lactic acid, pyruvic acid and nicotinamide adenine dinucleotide (NAD^+) were purchased from Shanghai Macklin Biochemical Co., Ltd. Sodium perchlorate monohydrate ($NaClO_4 \cdot H_2O$) was purchased from Sinpharm Chemical Reagent Co., Ltd. Pluronic P123 ($PEO_{20}PPO_{70}PEO_{20}$) and F127 ($PEO_{106}PPO_{70}PEO_{106}$) were supplied by Sigma-Aldrich. 2-aminoterephthalic acid (BDC-NH_2) was supplied by Alfa Aesar Chemicals. Acetic acid (AA) were purchased from Sinpharm Chemical Reagent Co., Ltd.. Alcohol dehydrogenase (ADH), lactate dehydrogenase (LDH) and diaphorase were obtained from Shanghai Aladdin Biochemical Technology Co., Ltd. All other chemicals were obtained from Shanghai Titan Scientific Co., Ltd.. All reagents were of analytical grade, and used without further purification. The applied water (18.1 $M\Omega \cdot cm^{-1}$) in the experiments was purified from a NW Ultrapure Water System (Heal Force, China).

2. Instruments and Methods

The powder X-ray diffraction (XRD) patterns were obtained on a Bruker D8 instrument using Cu Kα radiation (40 kV, 40 mA). The field emission scanning electron microscopy (FESEM) was conducted on Hitachi S-4800. Transmission electron microscopy (TEM) was conducted on a JEM-2100F electron microscope. N_2 sorption isotherms were recorded using a surface area and pore size analyzer (Micromeritics Tristar 3020). All of the samples were degassed under vacuum at 120 ^oC for 12 h prior to analysis. The specific surface area was calculated using the Brunauer-Emmett-Teller (BET) method using adsorption data at a relative pressure (P/P_0) lower than 0.15. The Barrett-Joyner-Halenda (BJH) pore size distributions were determined from the adsorption branches of the N_2 isotherms. Mercury porosimetry was measured on a AutoPore V 9600 analyser (Micromeritics, pressure range: 0.10 to 61000 psia). UV-Vis absorption spectra were measured with a UV-2550 spectrophotometer (Shimadzu, Tokyo, Japan). The fluorescent

spectra were measured with an RF-5301PC spectrofluorophotometer (Shimadzu). ^{1}H nuclear magnetic resonance (^{1}H NMR) spectra were carried out with a Bruker Avance III 400. Prior to ^{1}H NMR measurement, samples were digested at 80 °C for 2 h by adding 600 μL of a 1M NaOH in D_2O solution to a centrifuge tube containing 20 mg of sample. After the digestion, the inorganic component was removed by centrifugation and the supernatant was transferred into an NMR tube.

3. Experimental Section

NAD^+ immobilization: 10 mg DMUiO was dispersed in 2 mL water containing 2 mg NAD^+. The suspension was agitated at 25 °C for 1 h. The NAD^+-immobilized DMUiO was collected by centrifugation and washed with water for three times and dispersed in 2 mL H_2O for further use. The loading capacity was calculated by measuring the intensity variation of the absorbance of NAD^+ in the supernatant before and after loading.

Enzyme immobilization: 10 mg NAD^+@DMUiO was dispersed in 2 mL water containing 2 mg desired enzymes. The suspension was agitated at 25 °C for 1 h. The enzyme-immobilized NAD^+@DMUiO was collected by centrifugation and washed with water for three times and dispersed in 5 mL H_2O for further use. The loading capacity was calculated by BCA method.

Activity tests of ADH in DMUiO: All stock solutions were prepared with 50 mM HEPES buffer (pH 7.2). 100 μL NAD^+@ADH@DMUiO solution (2 mg/mL) was added into 2 mL HEPES buffer, then 30 μL ethanol was added, the emission intensity was measured at λ_{em} = 450 nm at 25 °C.

Activity tests of cascade reaction in DMUiO: All stock solutions were prepared with 50 mM HEPES buffer (pH 7.2). 100 μL NAD^+@LDH@Diaphorase@DMUiO solution (2 mg/mL) was added into 2 mL HEPES buffer, then 30 μL L-lactic acid (10 mM) and 50 μL resazurin (1 mM) was added. UV-Vis absorption spectra of the solution were recorded over 12 h.

Supplementary Table 1. Experimental parameters for the synthesis of UiO-66-NH_2 with different architetures described in this work.

Samples	P123 (mg)	F127 (mg)	P123/F127	Aromatic Agent	P123/F127/Aromatic Agent Mass Ratio
**Bowl-like UiO-66-NH_2**	50	25	2:1	TMB 120 μL	1/0.5/2.08
**Dendritic UiO-66-NH_2 (DMAUiO)**	35	40	7:8	Toluene 80-100 μL	1/1.14/1.99-2.49
	45	30	3:2		1/0.67/1.74-2.18
	50	25	2:1		1/0.5/1.39-1.74
	60	15	4:1		1/0.25/1.16-1.45
**Walnut-shaped UiO-66-NH_2**	75	0	1:0	Toluene 70 μL	1/0/0.81
**UiO-66-NH_2 crumpled sheet**	75	0	1:0	Toluene 90-100 μL	1/0/1.05-1.16
**UiO-66-NH_2 nanodisks**	75	0	1:0	Benzene 120 μL	1/0/1.41

Supplementary Figures

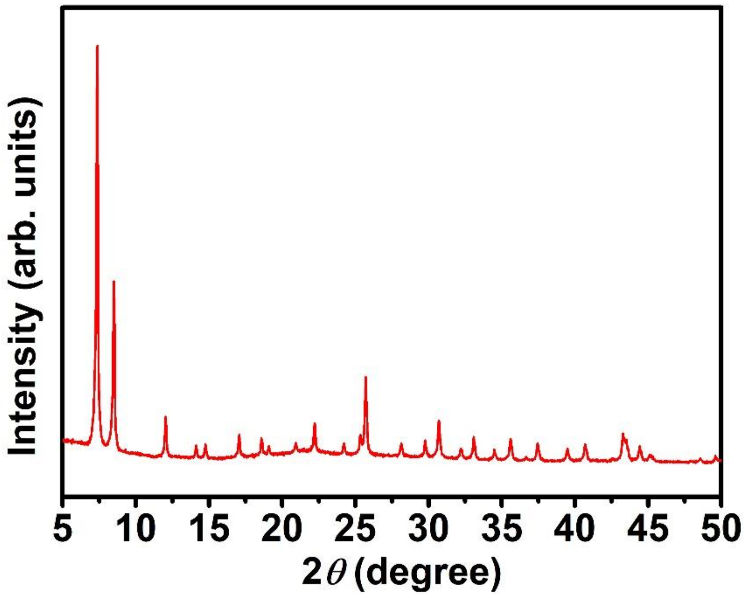

Supplementary Figure 1. XRD pattern of DMAUiO synthesized with P123/F127 mass ratio of 2:1 and 80 μL toluene.

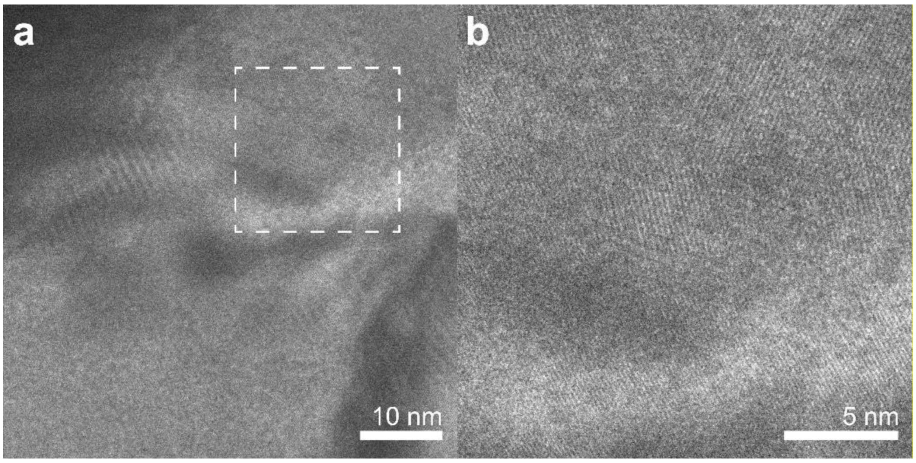

Supplementary Figure 2. (**a**) A typical HRTEM image of DMAUiO. (**b**) Enlarged image of the heighted area shown in (a).

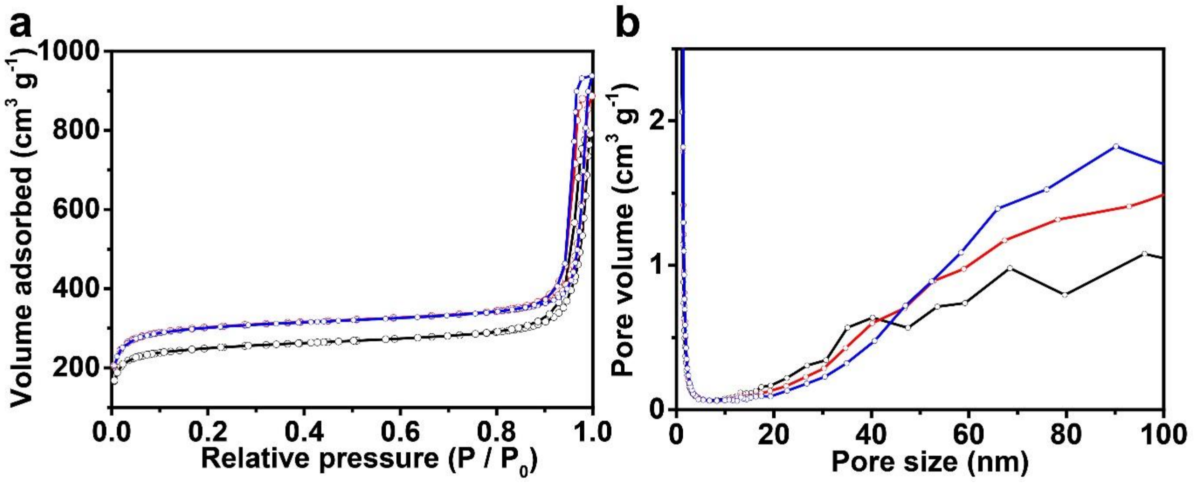

Supplementary Figure 3. N_2 sorption isotherms (**a**) and the corresponding pore size distribution (**b**) for DMAUiO synthesized with 60 μL (black), 80 μL (red) and 100 μL (blue) toluene when the P123/F127 feed ratio is 2:1. The surface area of each sample is about 956 (60 μL), 1153 (80 μL) and 1148 m^2 g^{-1} (100 μL), respectively.

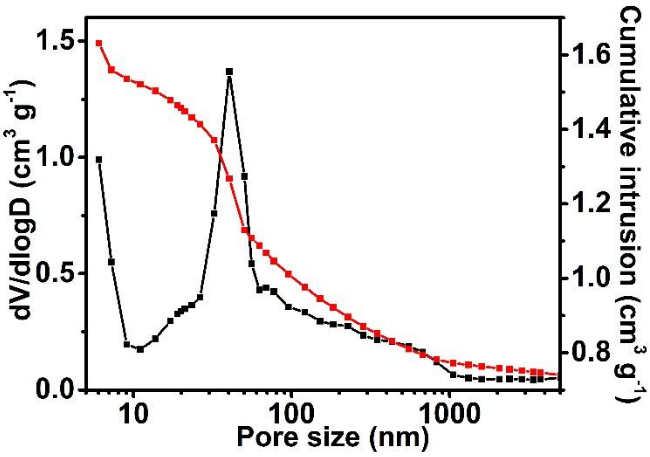

Supplementary Figure 4. Cumulative pore volume curve (red) and pore size distribution (black) derived from mercury porosimetry analysis for the sample of DMAUiO synthesized with 80 μL toluene when the P123/F127 feed ratio is 2:1.

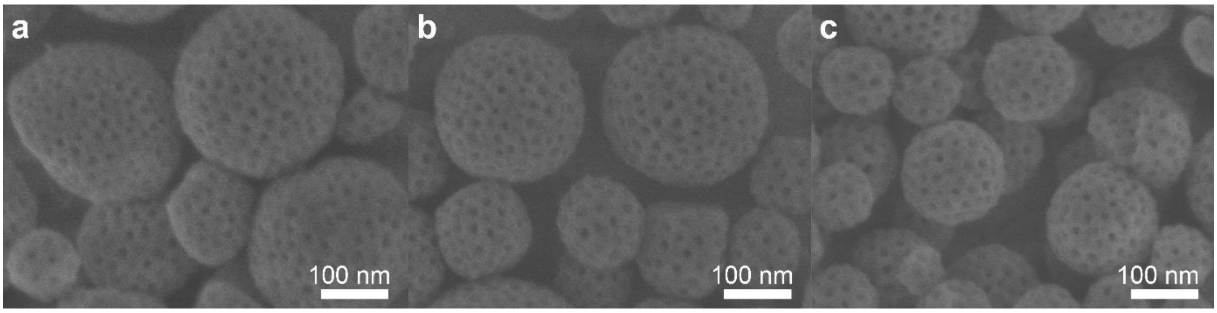

Supplementary Figure 5. SEM images of MOFs synthesized with F127 and 60 μL (**a**), 80 μL (**b**) and 100 μL (**c**) toluene.

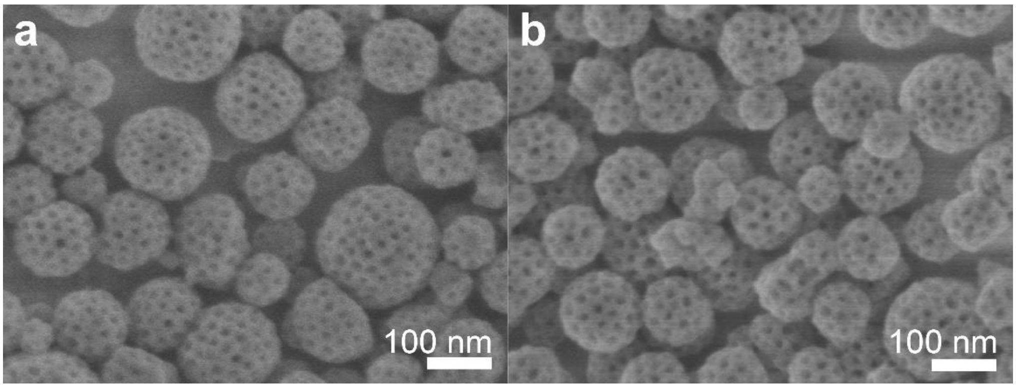

Supplementary Figure 6. SEM images of MOFs synthesized with 60 μL (**a**) and 100 μL (**b**) toluene when the P123/F127 feed ratio was set at 1:2.

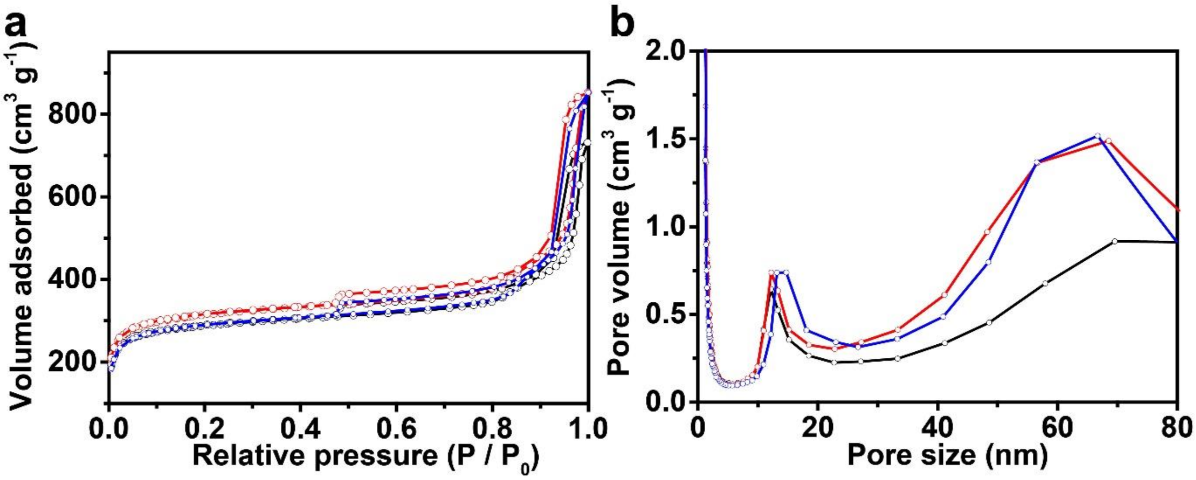

Supplementary Figure 7. N_2 sorption isotherms (**a**) and the corresponding pore size

distribution (**b**) for DMAUiO synthesized with 60 μL (black), 80 μL (red) and 100 μL (blue) toluene when the P123/F127 feed ratio was set at 1:2. The surface area of each sample is about 1097 (60 μL), 1194 (80 μL) and 1104 $m^2\ g^{-1}$ (100 μL), respectively.

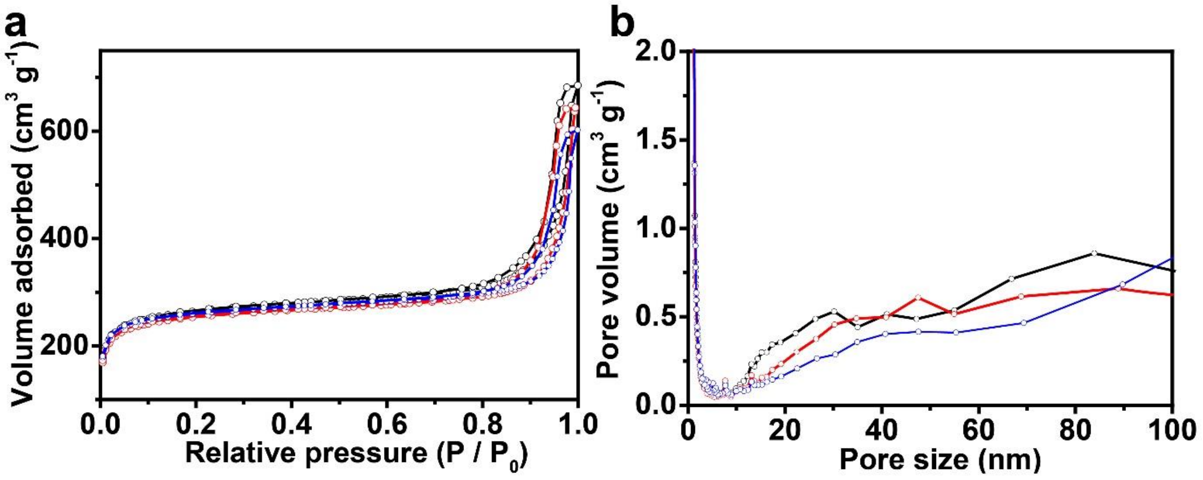

Supplementary Figure 8. N_2 sorption isotherms (**a**) and the corresponding pore size distribution (**b**) for DMAUiO synthesized with 60 μL (black), 80 μL (red) and 100 μL (blue) toluene when the P123/F127 feed ratio was set at 7:8. The surface area of each sample is about 1001 (60 μL), 962 (80 μL) and 986 $m^2\ g^{-1}$ (100 μL), respectively.

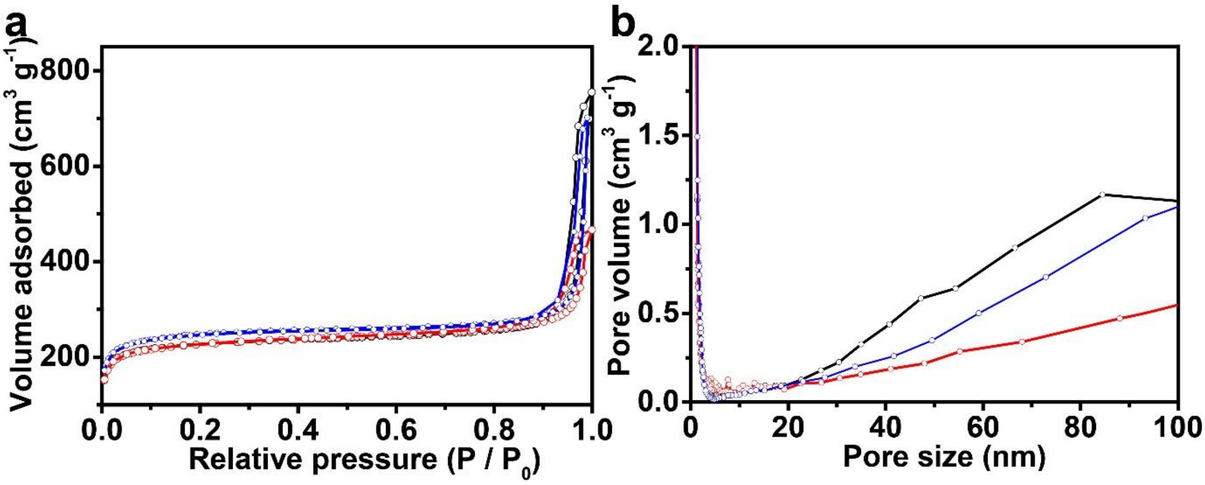

Supplementary Figure 9. N_2 sorption isotherms (**a**) and the corresponding pore size distribution (**b**) for DMAUiO synthesized with 60 μL (black), 80 μL (red) and 100 μL (blue) toluene when the P123/F127 feed ratio was set at 4:1. The surface area of each sample is about

860 (60 μL), 862 (80 μL) and 950 m^2 g^{-1} (100 μL), respectively.

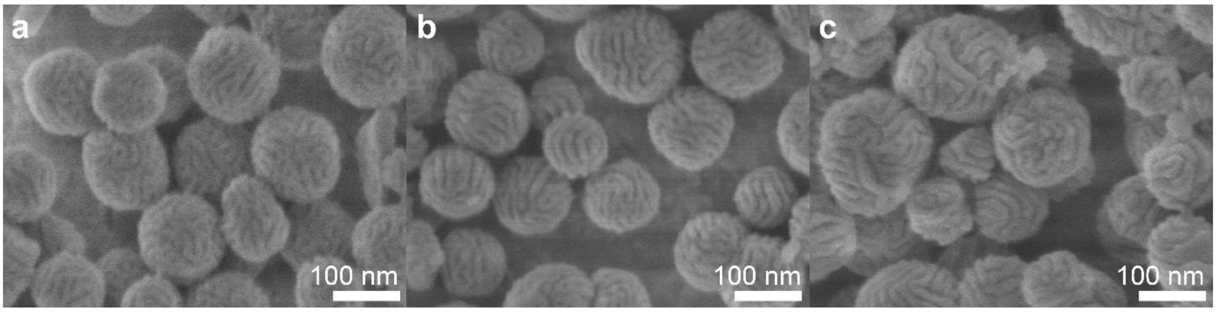

Supplementary Figure 10. SEM images of MOFs synthesized with 0 μL (**a**), 10 μL (**b**) and 20 μL (**c**) toluene when the P123/F127 feed ratio was set at 2:1.

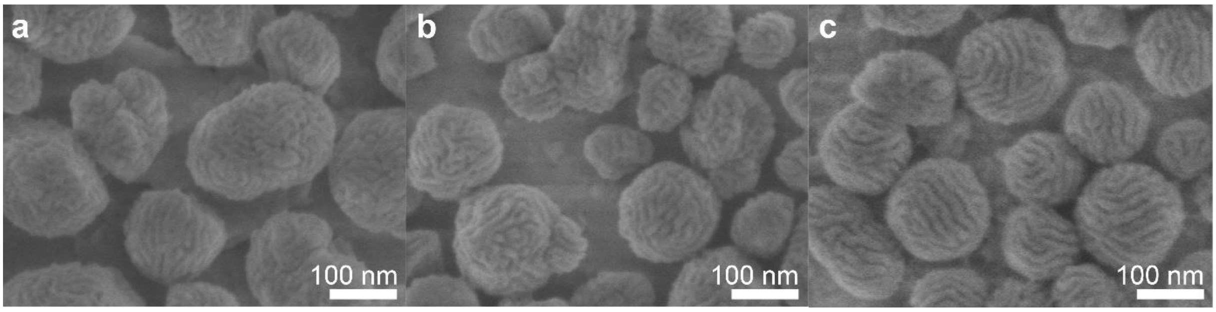

Supplementary Figure 11. SEM images of MOFs synthesized with 0 μL (**a**), 10 μL (**b**) and 20 μL (**c**) toluene when the P123/F127 feed ratio was set at 4:1.

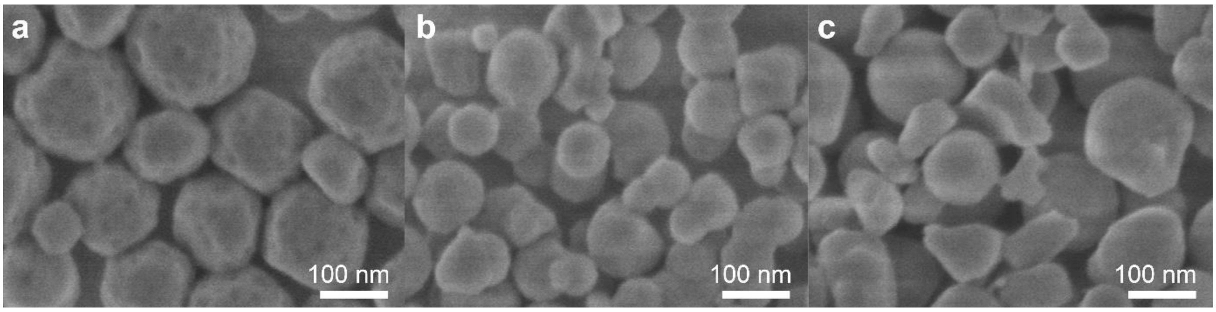

Supplementary Figure 12. SEM images of MOFs synthesized without (**a**) ClO_4^-, (**b**) surfactants, and (**c**) both ClO_4^- and surfactants.

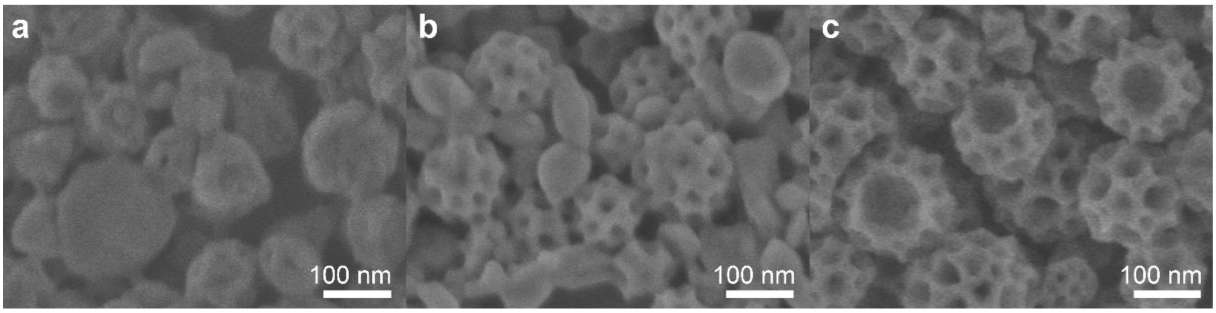

Supplementary Figure 13. SEM images of MOFs synthesized with 30 μL (**a**), 50 μL (**b**) and 100 μL (**c**) TMB when the P123/F127 feed ratio was set at 2:1.

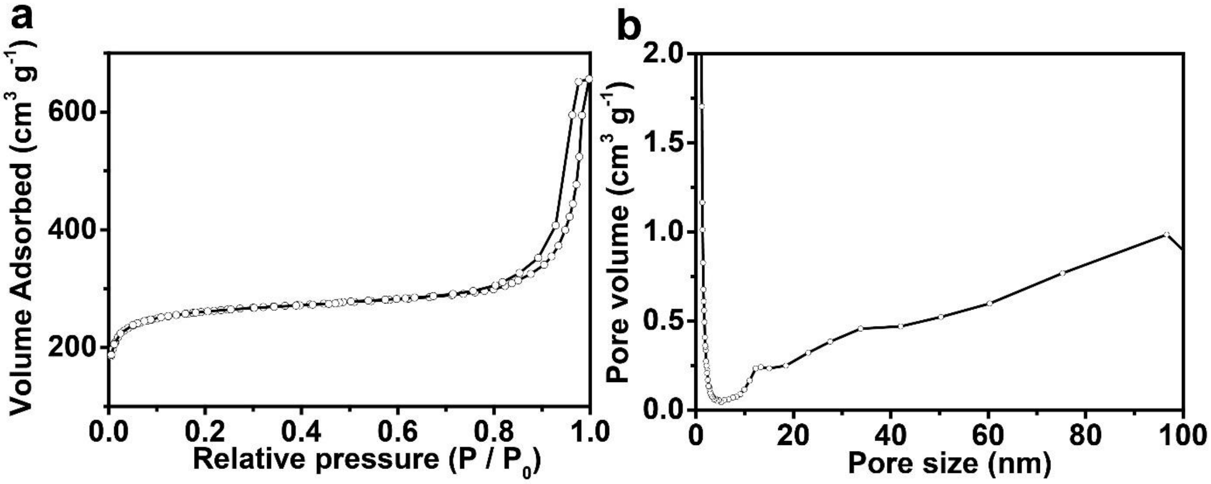

Supplementary Figure 14. N_2 sorption isotherm (**a**) and the corresponding pore size distribution (**b**) for bowl-like UiO-66-NH_2. The surface area of the sample is about 1189 m^2 g^{-1}.

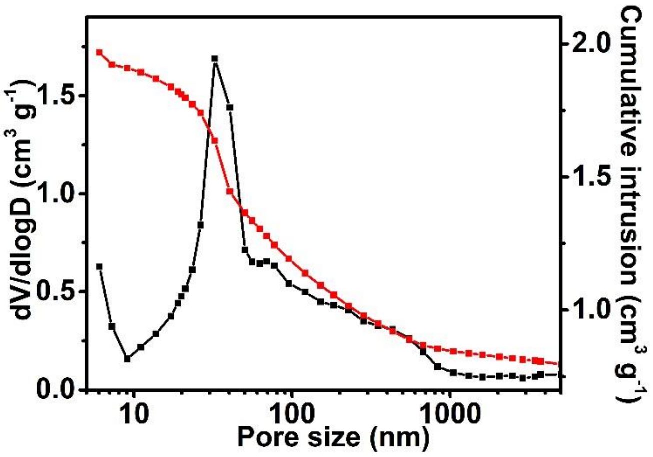

Supplementary Figure 15. Cumulative pore volume curve (red) and pore size distribution (black) for the sample of bowl-like UiO-66-NH_2 by mercury porosimetry analysis.

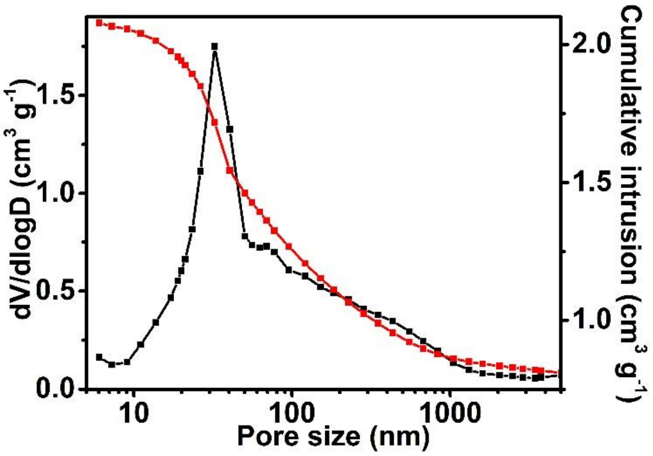

Supplementary Figure 16. Cumulative pore volume curve (red) and pore size distribution (black) for the sample of walnut-shaped UiO-66-NH_2 by mercury porosimetry analysis.

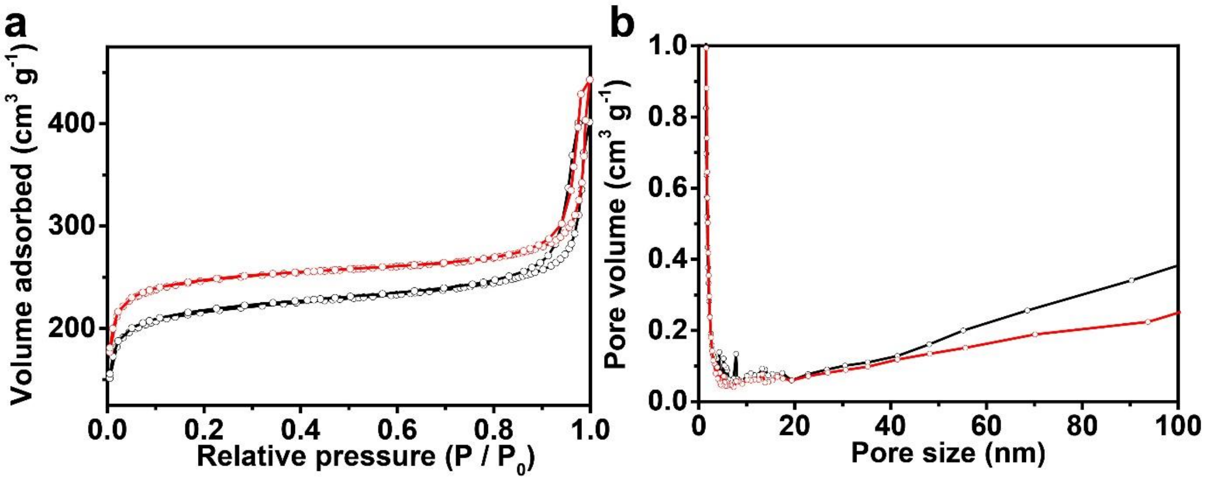

Supplementary Figure 17. N_2 sorption isotherms (**a**) and the corresponding pore size distribution (**b**) for hierarchical UiO-66-NH_2 synthesized with P123 and 70 μL (black) and 90 μL (red) toluene. The surface area of each sample is about 819 (70 μL) and 939 m^2 g^{-1} (90 μL), respectively.

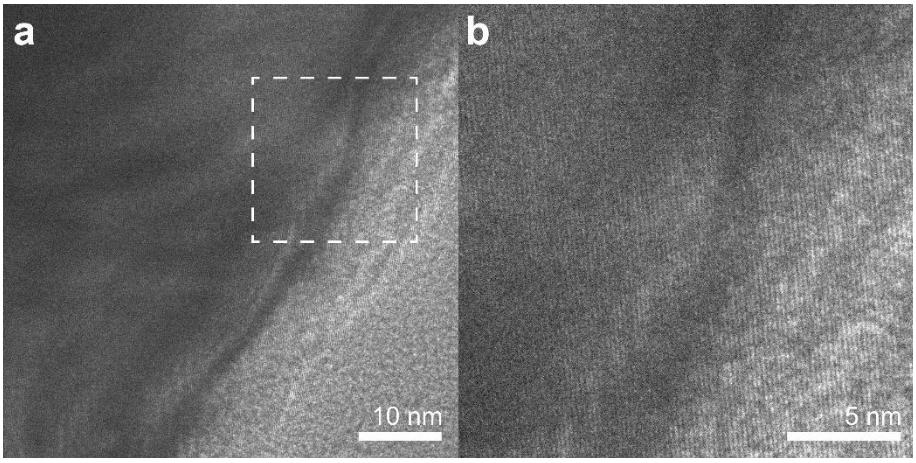

Supplementary Figure 18. (**a**) A typical HRTEM image of walnut-shaped UiO-66-NH_2. (**b**) Enlarged image of the heighted area shown in (a).

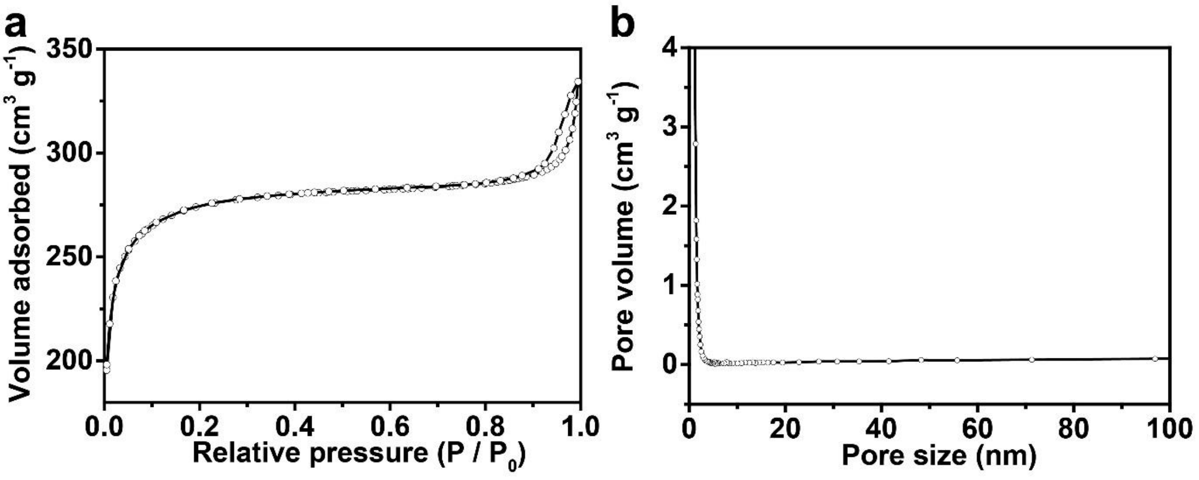

Supplementary Figure 19. N_2 sorption isotherm (**a**) and the corresponding pore size distribution (**b**) for UiO-66-NH_2 nanodisks synthesized with P123 and 120 μL benzene. The surface area of the sample is about 1050 m^2 g^{-1}.

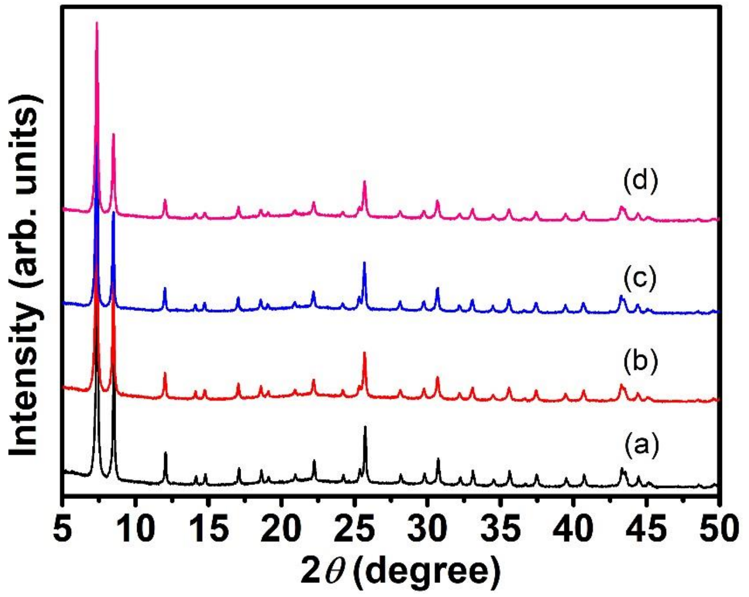

Supplementary Figure 20. XRD patterns of (**a**) bowl-like, (**b**) walnut-shaped, (**c**) crumpled nanosheet and (**d**) nanodisk UiO-66-NH_2.

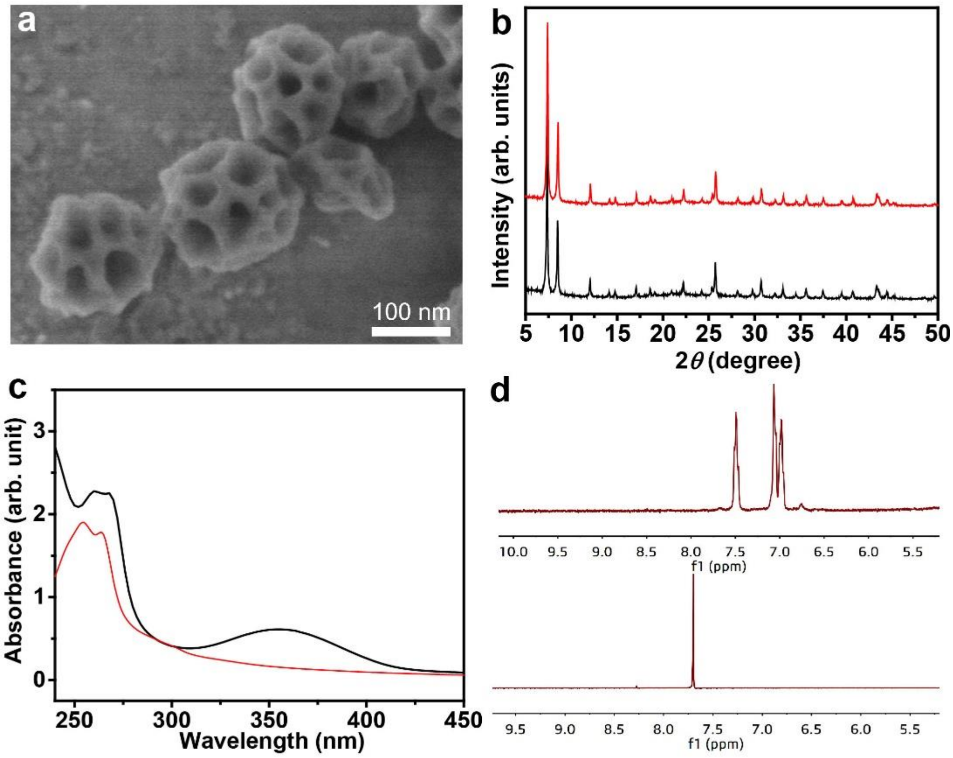

Supplementary Figure 21. (**a**) SEM image of DMUiO. (**b**) XRD patterns of DMAUiO before (black) and after (red) deamination. (**c**) UV-Vis spectra of the DMAUiO (black) and DMUiO (red). (**d**) ^{1}H NMR spectra of digested DMUiO (bottom) and DMAUiO (top).

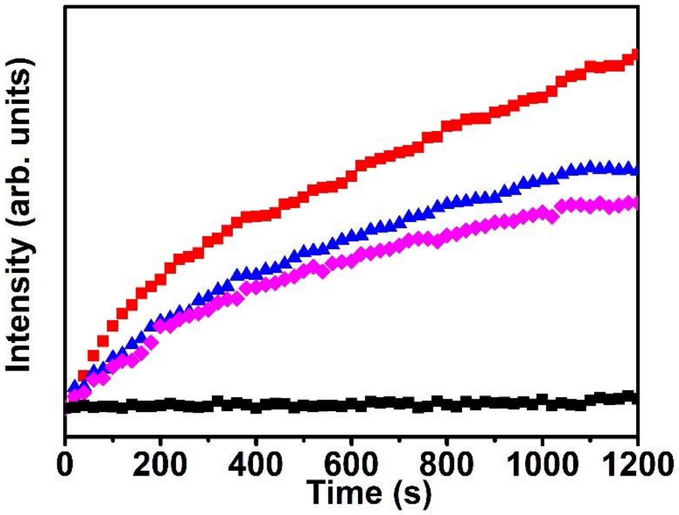

Supplementary Figure 22. Time-dependent fluorescence changes of different reaction system after the addition of ethanol: NAD^+@ADH@DMUiO-40 (red), NAD^+@ADH@DMUiO-20 (blue), free NAD^+/ADH system (pink) and ADH@DMUiO-40 (black).

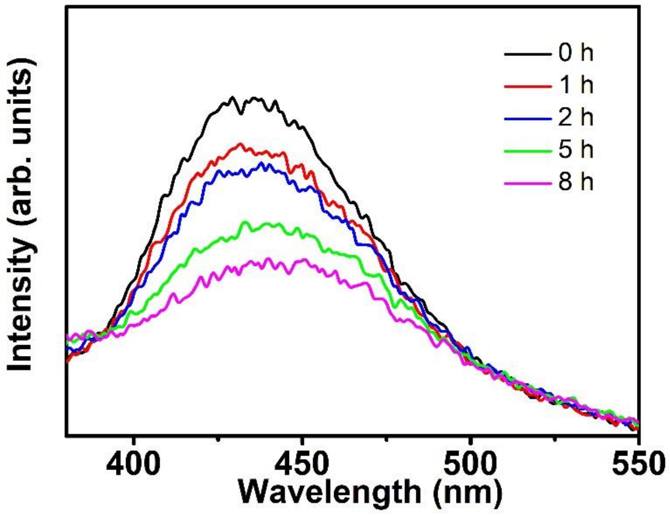

Supplementary Figure 23. Fluorescence spectra of the separated NAD^+@ADH@LDH@DMUiO system after the addition of pyruvic acid.

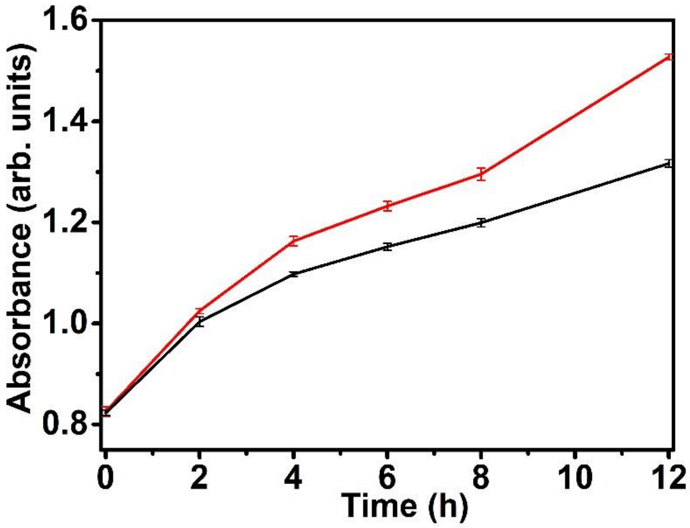

Supplementary Figure 24. Time-dependent UV-Vis absorption changes of LDH@diaphorase@NAD^+@DMUiO system (red) and free LDH/diaphorase/NAD^+ system (black) at 571 nm. Mean values and error bars are defined as mean and s.d., respectively.
